# Design, transform and control of optical field in discrete optical system: an example

**DOI:** 10.1038/s41598-017-05414-w

**Published:** 2017-07-12

**Authors:** Hongchang Deng, Yonggui Yuan, Libo Yuan

**Affiliations:** 1 Photonics Research Center, School of Electronic Engineering and Automation, Guilin University of Electronics Technology, Guilin, 541004 People’s Republic of China; 2 Key Laboratory of In-Fiber Integrated Optics, Ministry of Education, College of Science, Harbin Engineering University, Harbin, 150001 People’s Republic of China

## Abstract

A discrete optical system can broaden the spatial distribution of the input light through optical coupling in array waveguides, just like diffraction in continuous media. Here, we theoretically demonstrate several kinds of control methods of optical field propagation in a discrete optical system, which is composed of an Airy fiber with two perpendicular arrayed cores. A brief transform mechanism between Gaussian and Airy beam propagation in such a fiber is presented. The wavefront of the output beam from the Airy fiber is actually dependent on the phased arrayed modulation of coupling array cores. Except the optical wavelength changing, we propose two new methods, including fiber length and bending-induced refractive-index changing, to accomplish that modulation. The calculation results show that these new methods are very effective for the Airy phase modulation. By combining these methods and controlling the corresponding parameters, the Gaussian beam, the one-dimension Airy beam, and the two-dimension Airy beam can be obtained by one same Airy fiber. These methods are also generally applicable to the other discrete optical system and can be extended to generate any other types of optical beams, such as Bessel beams and Mathieu beams.

## Introduction

The Airy beam (AB) have propagation-invariant intensity profile and exhibit ‘self-healing’ features^1–3^. In particular, such a beam has an ability to remain transverse accelerating during propagation^4^, which moves on a parabolic trajectory very much like a body under the action of gravity. Owing to these unique properties, Airy beams have found many potential applications, such as optical micro-manipulation^5, 6^, imaging technology^7^, surface plasmon polaritons^8, 9^ and laser micromachining^10^. The direct generation of the Airy beam requires combining phase and amplitude modulation by imposing a cubic phase on a Gaussian beam and then taking its Fourier transform using a lens. Several AB generations and controls using bulk optic configuration were reported, such as spatial light modulator (SLM)^1, 11^, continuous phase mask^12^, nonlinear photonic crystal^13, 14^, and optically induced refractive index gradient^15^. In our previous work^16, 17^, a one/two-dimensional (1D/2D) Airy-like beam technique was reported based on array waveguides instead of a cubic phase plate. Such an Airy fiber has a small size, easy handling, stable and strong anti-jamming capability.

In the discrete optical system, waveguide arrays have been used to engineer the diffraction properties of optical wave fronts^18^. In this paper, we study theoretically the propagation properties of light in a discrete optical system of the Airy fiber with a 2D arrayed-core. We focus on studying the transform mechanism between Gaussian and Airy beam propagation in such a fiber. To control the propagation dynamics of light in the fiber, several modulation methods are introduced by changing the controlling parameters. We further demonstrate the consistency and validity of these methods and reveal that the propagation properties of Airy fiber are strongly related to the phased array modulation based on waveguide coupling.

## Results

### The mode coupling in a straight or bent Airy fiber

**Figure 1:**
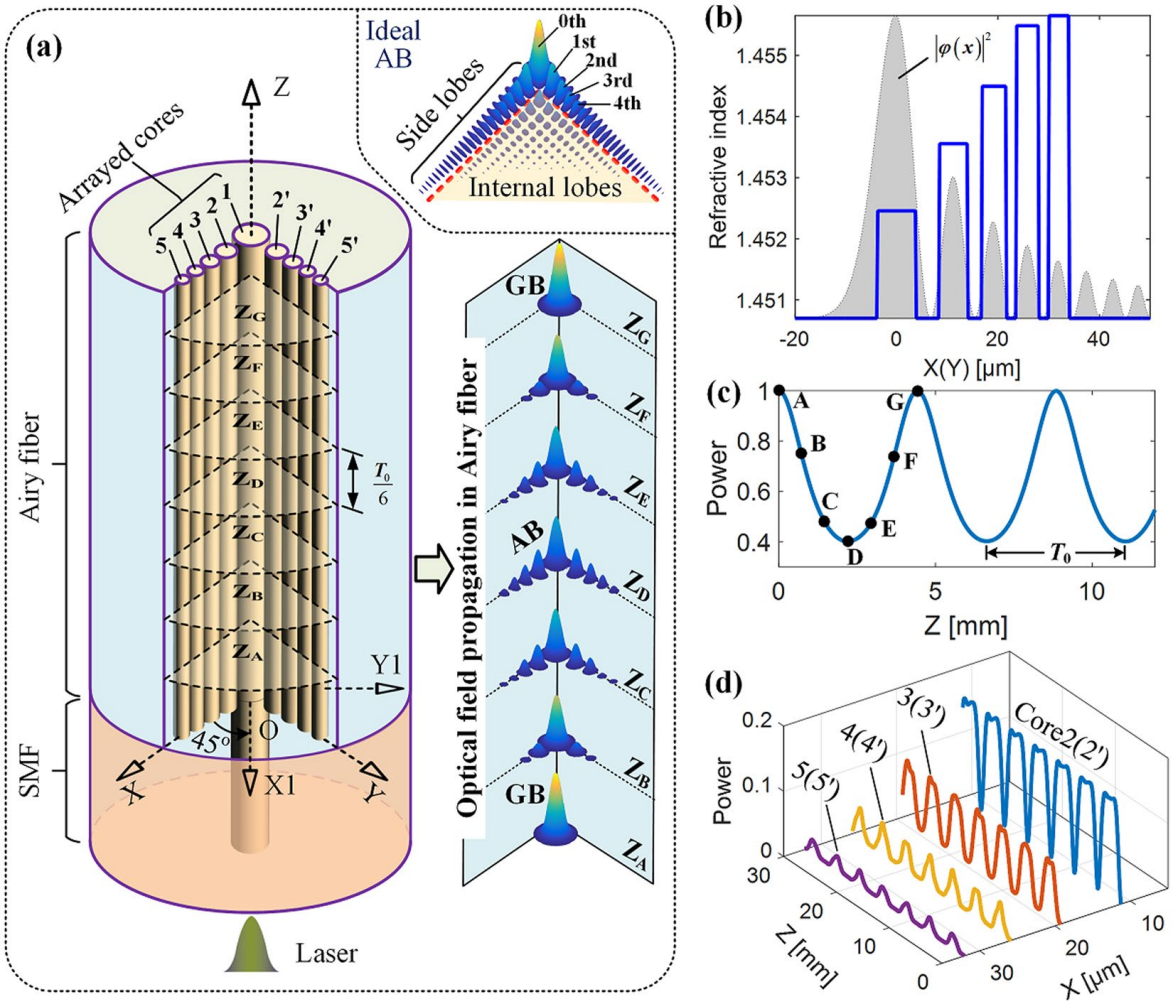
Wave propagation in an Airy fiber. (**a**) Working principle (left) and the transverse fields propagation in the fiber (right). The inset is corresponding the ideal Airy beam. (**b**) The RI profile (solid line). The shaded area in (**b**) depicts the Airy intensity pattern. (**c** and **d**) are normalized power curves as a function of fiber length in the center main core and side cores, respectively.

The finite energy Airy beam distribution is given by the inset of Fig. 1(a). We note that most of the beam energy is carried by the L-shaped side lobes. Therefore, an arrayed-core fiber could be used to generate the L-shaped beam, which can retain most of the characteristics of the ideal Airy beam. Considering space limitations, we have to arrange a limited number of cores in a fiber. Thus, we propose an Airy fiber with nine cores to generate that Airy-like beam with the main lobe (0^th^) and the outermost 1^st^ to 4^th^ order side lobes, as shown in Fig. 1(a). There are two perpendicular arrayed cores arranged in the x-axis (namely, core 1, 2, 3, 4, 5) and the y-axis (namely, core 1, 2′, 3′, 4′, 5′) embedded in a common cladding. The main core 1 is located at the center of the fiber, where the origin *O* of the Cartesian coordinate system (X, Y, Z) is taken to be. To observe optical field along a 45-degree direction, another coordinate system (X1,Y1, Z) is also established by rotating the (X, Y, Z) coordinate system 45 degrees clockwise around the z-axis. The refractive-index (RI) distribution of the arrayed-core is represented by the solid line in Fig. 1(b) (see the supplementary material). In order to ensure the stability of the propagating wave, every core of the fiber can only support one mode. And the center of every core of the fiber is located at the intensity peaks of corresponding optical lobes of Airy field |*ψ*(*x*)|^2^ from equation (2) for *λ* = 980 nm, *m*_0_ = 5 μm, *m*_1_ = 4.79 μm, *a*_*m*_ = 0.06, *v*_*m*_ = 0, *y* = z = 0. Moreover, a Gaussian beam (GB) is coupled into the center main core of the Airy fiber by fusion splicing a single mode fiber (SMF), as shown in Fig.1(a).

Based on the supermode theory in arrayed-waveguide (see eq. (12)), we calculate the optical coupling power in the main core and side cores of the Airy fiber while Gaussian beam inputs, as shown in Fig. 1(c) and (d). One can find that the magnitude of the coupling power decreases gradually from inside out. And the light coupling among arrayed-core is periodical, whose coupling period *T*_0_ is 4.4 mm depicted in Fig. 1(c). Figure 1(a) also shows the transverse field patterns (left side) at different fiber lengths Z = (0.5 + *m*/6)*T*_0_. Here *m* is any integers among −3 to 3, that is corresponding to fiber lengths Z_A_, Z_B_, …, Z_G_, respectively. In the first half period (from Z_A_ to Z_D_), as fiber length increases, the intensity of propagating light in the center main core is decreased and at the meantime side cores’ are increased. In the second half period (from Z_D_ to Z_G_), light powers in side cores are gradually coupled into the center main core with fiber length increasing and finally one can obtain a Gaussian-like beam at Z_G_. Consequently, the Gaussian-like beam and the Airy-like beam can be converted each other using a half period *T*_0_/2 length of the Airy fiber. And that means, as a Gaussian beam input, one can obtain an Airy-like beam and a Gaussian-like beam with (2 *m* − 1)*T*_0_/2 and *mT*_0_ length of Airy fiber (*m* is any positive integer), respectively.

**Figure 2:**
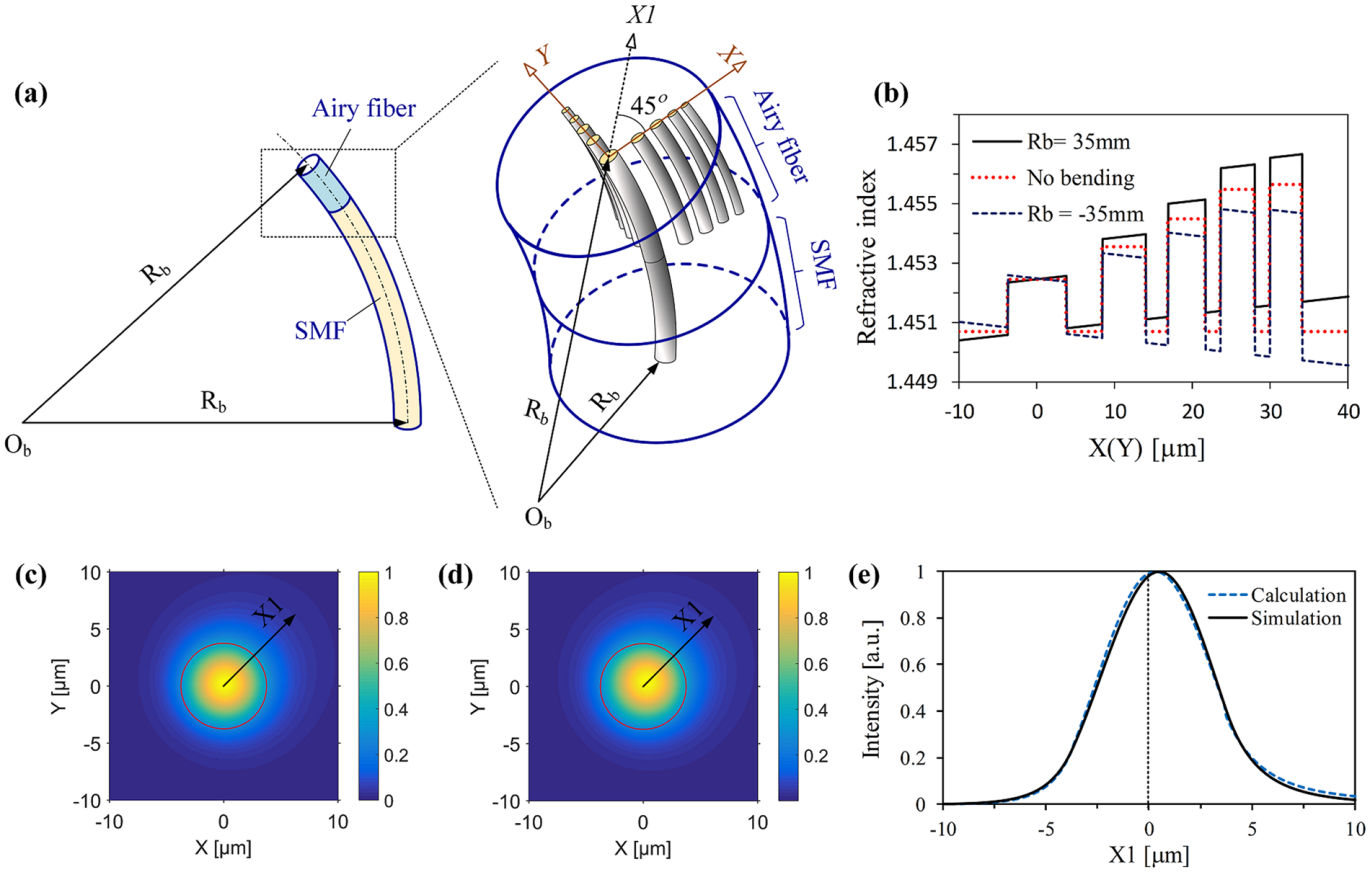
Bent Airy fiber. (**a**) The schematic diagram of bending an Airy fiber connected an SMF with a bending radius *R*_*b*_. The bending radius of the fiber along the positive/negative X1-axis direction is set to positive/negative value. (**b**) The RI distributions of the arrayed-core with a bending radius of 35 mm (solid line) and −35 mm (dashed line), and without bending (dotted line) along the X(or Y) axis. (**c** and **d**) are the calculation and simulation mode fields in Core 1, respectively. (**e**) is corresponding to the intensity profiles of fields in (**c** and **d**) along the X1-axis.

Figure 2(a) shows a schematic diagram of a bent Airy fiber. Note that the light propagation in the arrayed-core of the bent Airy fiber is sensitive to the magnitude and direction of the bending radius due to its asymmetry structure. Therefore, for the sake of simplicity, the bending direction of the Airy fiber is assumed along the X1-aixs. In this case, we give the equivalent RI distribution of bent Airy fiber using eq. (15), as shown in Fig. 2(b). From the figure, we can find that the equivalent RI distribution of bent fiber is tilted with respect to the original without bending. And the corresponding linear change can be determined by the magnitude and direction of the bending. Figure 2(c) gives the calculation result of the transverse mode field propagation in Core 1 with a bending radius of 35 mm using eq. (13). We can clearly see that the mode field shifts away from the core center, which causes a distortion along the bending direction. For comparison, the simulation results are obtained by using commercial COMSOL Multiphysics software. From Fig. 2(c)–(e), we can find that the calculation results are very agree with the simulation results.

### Transform mechanism in the Airy fiber

**Figure 3:**
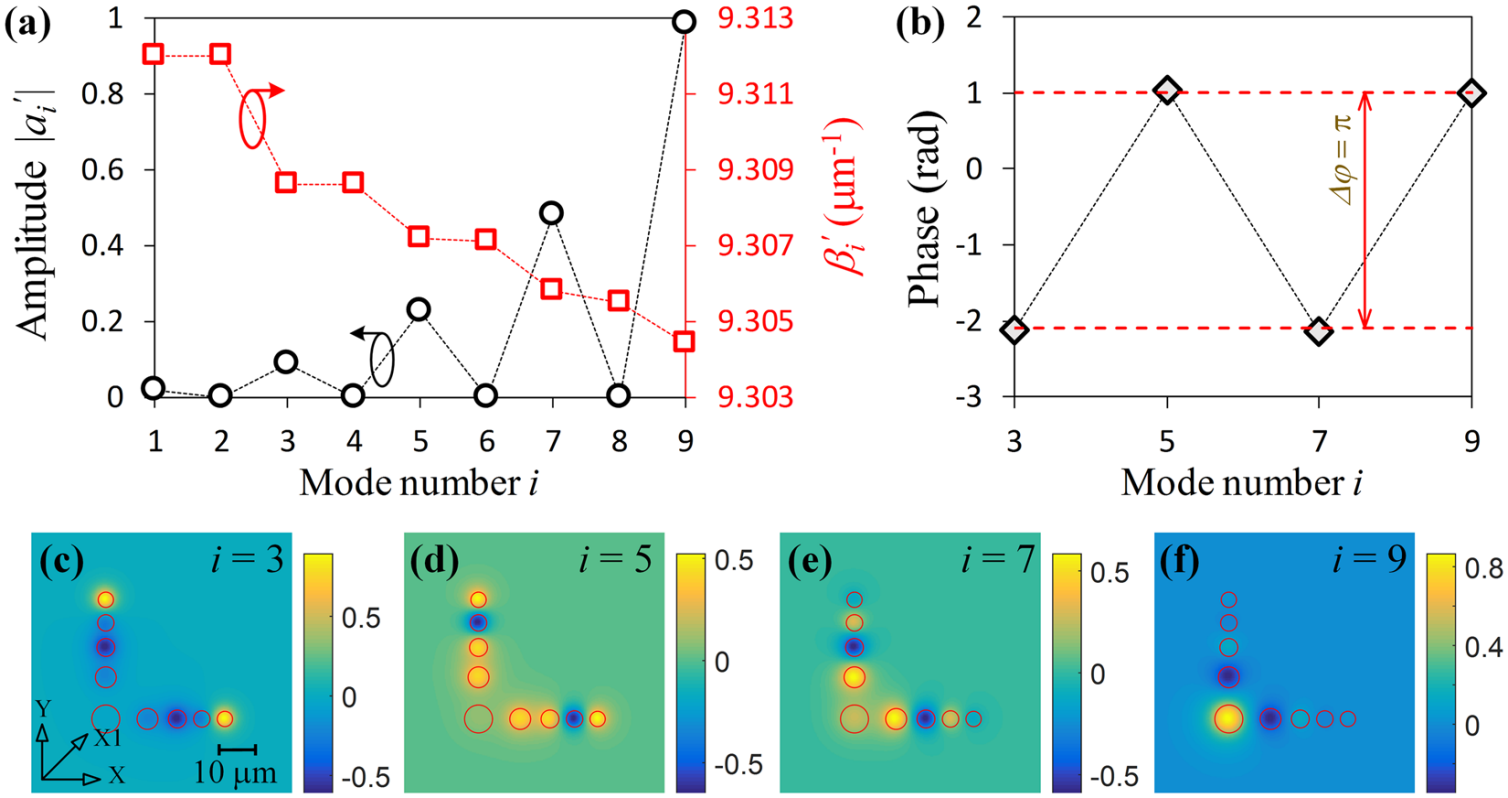
Supermode properties of the Airy fiber. (**a**) The modal amplitude ${a}_{i}^{^{\prime} }$ and the propagation coefficient ${\beta }_{i}^{^{\prime} }$ as a function of mode number *i*. (**b**) Phases of four excited supermodes at Z = (2 *m* − 1)*T*_0_/2. (**c**–**f**) Supermode patterns with 3-, 5-, 7-, and 9-order, respectively.

**Figure 4:**
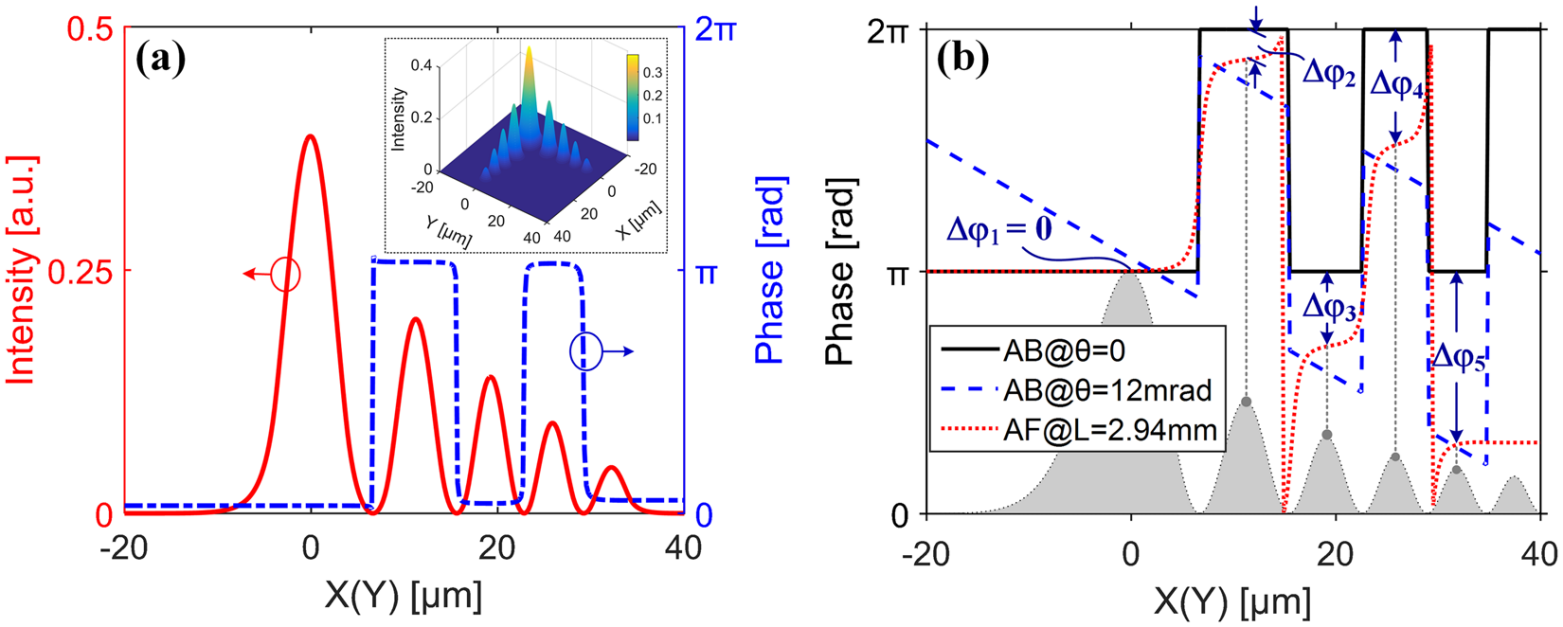
The properties of the output beam from the Airy fiber. (**a**) The intensity and phase distributions of the output beam from Z_D_ = 2.2 mm (*T*_0_/2) length of the Airy fiber. The solid line and the dash-dot line depict intensity and phase distributions in arrayed cores along the x-axis or the y-axis, respectively. The inset is corresponding transverse intensity pattern.(**b**) The phase curves of the output beam from Z_E_ ≈ 2.94 mm (*T*_0_/2 + *T*_0_/6) length of Airy fiber (dot line) and the ideal Airy beams with launch angles θ = 0 (solid line) and θ = 12 mrad (dash line). The shaded area in (**b**) is corresponding to the optical intensity profile of the ideal Airy beam with launch angles θ = 0.

Using the coupling matrix *M* in eq. (9), we can calculate the propagation constants ${\beta }_{i}^{^{\prime} }$ of the guided supermodes in the arrayed-core of the Airy fiber, which are given by dashed line marked circle in Fig. 3(a). One can find that there are four pairs of degenerate modes whose mode number are 2*m*−1 and 2 *m* (*m* = 1, 2, 3, 4). However, not all supermodes can be excited in Airy fiber. Based on the absolute values of mode amplitude depicted by the dashed line marked square in Fig. 3(a), we can see that mainly excited supermodes are 3-, 5-, 7-, 9-order modes, especially the 9-order mode which contains a large percentage of the total power (more than 80% in our case). The transverse intensity patterns of mainly excited supermodes are shown in Fig. 4(c)–(f). Note that all the mode fields are symmetric to the X1-axis for Gaussian beam input.

As mentioned above, we have demonstrated that an Airy-like beam can be generated by a (2 *m* − 1)*T*_0_/2 length of Airy fiber after periodical amplitude and phased arrayed modulation through supermodes interference. In Fig. 4(a) and (b), we can find that there is almost no phase difference between such the Airy-like beam and the ideal Airy beam with the launch angle *θ* = 0. However, the additional phase should be introduced into the Airy-like beam if we change the length of the Airy fiber. In Fig. 4(b), one can find that the phase differences (Δ*φ*_1_, Δ*φ*_2_, …, Δ*φ*_5_) between the two beams are increased from the main lobe to side lobes, just like an ideal Airy beam with the launch angle *θ* = 12 mrad.

From eq. (11), we can find that the modal amplitude ${\tilde{a}}_{i}^{^{\prime} }$ (see Fig. 3(a)) and field distributions ${{\boldsymbol{E}}}_{i}^{^{\prime} }$ (see Fig. 3(c)–(f)) can provide Airy amplitude modulation, so that Airy-like intensity profiles can be obtained along the fiber arrayed-core, as shown in Fig. 1(a). However, how to obtain Airy phase modulation (see Fig. 4(a))? The different propagation constants ${\beta }_{i}^{^{\prime} }$ of excited supermodes during propagation in fiber arrayed-core can induce phase differences. Thus, a phased array is formed in a discrete optical system of the Airy fiber. We can change parameters of the fiber arrayed-core to control the phased array. Particularly, in Fig. 3(b), we design a special arrayed cores (see Fig. 1(a) and (b)) to form a phased array whose phase differences between two adjacent excited supermodes are just *π* at Z = (2 *m* − 1)*T*_0_/2, just like cubic phase modulation with a Gaussian beam in the process of the ideal Airy beam generation. Thus, by the supermodes interference along the arrayed-core of the (2 *m* − 1)*T*_0_/2 length of the Airy fiber, both Airy amplitude and phased array modulation with a Gaussian beam are implemented to generate a 2D Airy-like beam, which almost has the same amplitude and phase distributions of ideal Airy beam, as shown in Fig. 4(a) and (b). Remarkably, to ensure the stable and high-quality beam output, a short Airy fiber (a few millimeters length in our case) is chosen by us.

### Properties of the output beam from different length of Airy fiber

**Figure 5:**
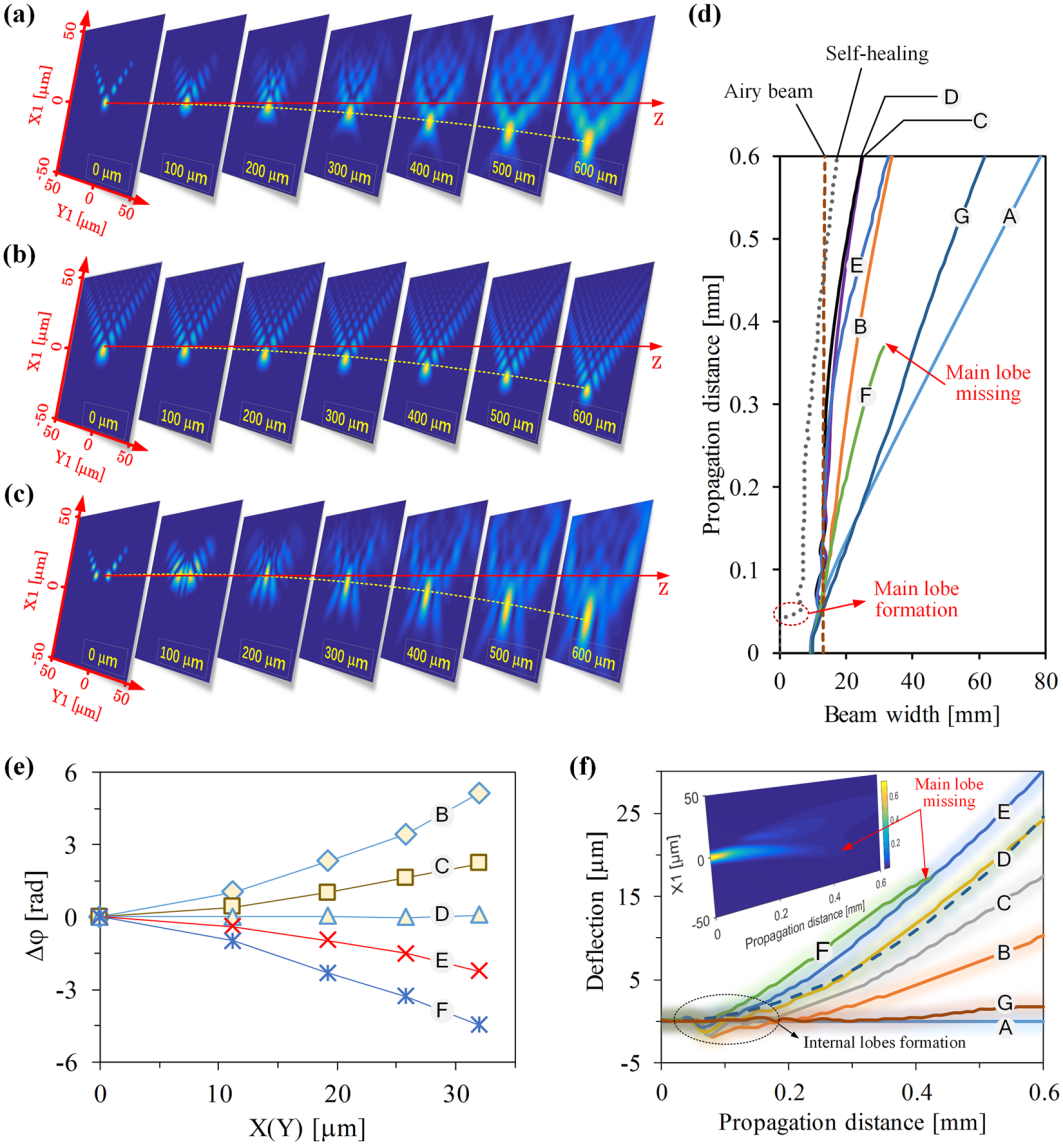
Properties of the output Airy-like beams from different lengths of the Airy fiber. (**a**) The normalized transverse propagation patterns of the output beam from Z_D_ = 2.2 mm length of the Airy fiber. (**b**) The corresponding ideal Airy beam propagation patterns with the same parameters (*m*_0_ = 5 μm, *m*_1_ = 4.79 μm, *a*_*m*_ = 0.06, *θ*_*m*_ = 0). **(c)** The self-healing of the Airy-like beam in (**a**) when its main lobe is blocked. (**d**) The main lobe widths of the propagating beams along the Y1-axis. (**e**) The additional phase differences at peak locations of the generated Airy-like beams compared with the ideal Airy beam. (**f**) The corresponding deflection (solid lines) of the main lobe of the generated beams during propagation. The data marked A is corresponding to the input Gaussian beam. And the data marked B, C, D, E, F, and G are corresponding to the output beams from the Airy fibers with lengths Z_B_, Z_C_, Z_D_, Z_E_, Z_F_, and Z_G_, respectively. The dashed lines in (**d** and **f**) are corresponding to the ideal Airy beams. The inset in **(f)** indicates the propagation dynamics of the generated Airy-like beam from Z_F_ = 3.68 mm length of the Airy fiber.

Figure 5(a) shows the transverse patterns of the generated Airy-like beam during propagation in free space. Here the calculated results are based on the angular spectrum method^19, 20^. For simplicity, the origin coordinate is located at the fiber end-face for observing the propagation behavior of the generated Airy-like beam from the Airy fiber. We can clearly see that the internal lobes of the Airy-like beam are gradually reborn during propagation due to self-healing, just like a complete ideal 2D Airy beam (see Fig. 5(b)). Furthermore, if we block the main lobe of the Airy-like beam, this incomplete Airy-like beam also shows an extraordinary self-healing property to form its main lobe after propagating dozens of micrometers, as shown in Fig. 5(c). In the Y1-axis direction, the width of the main lobe of the incomplete Airy-like beam is always less than the ideal Airy beam during short-range propagation (hundreds of micrometers), as shown in dotted and dashed lines of Fig. 5(d). However, in the X1-axis direction, the width of the beam’s main lobe can not remain invariant compared with the ideal Airy beam during propagation due to lack of the internal lobes, as shown in Fig. 5(b) and (c).

As mentioned above, an Airy-like beam or a Gaussian-like beam can be obtained by changing Airy fiber length. Therefore, the corresponding output beam properties for a certain length of the Airy fiber, especially the width of the main lobe, could be similar to Airy beam or Gaussian beam. From Fig. 5(d), we can find that the generating Airy-like beams for the Airy fiber length near to (2 *m* − 1)*T*_0_/2 remain almost nonspreading during short-range propagation and the widths of their main lobes along the Y1-axis can keep almost invariant compared with the ideal Airy beam, as shown in the lines of Fig. 5(d) marked C, D, and E. However, the output beams from the Airy fibers with a length near to *mT*_0_ are more like Gaussian beams so that there are similar changing trends of the widths of their main lobes, as shown in the lines of Fig. 5(d) marked A, B, F, and G.

From Fig. 5(a), we can find that the Airy-like beam has a remarkable ability to accelerate its main lobe along the direction of the negative X1-axis (see the trajectory marked with the dashed line in Fig. 5(a)). Actually, the transverse shift of the Airy beam depends on the initial launch angle *θ*_*m*_, which is determined by the phase changing trend compared with an ideal Airy beam with *θ*_*m*_ = 0^21, 22^. In our case, the slope of the linear fitting curve of the additional phase differences as an equivalent launch angle can evaluate such phase changing trend^23^. From Fig. 5(e), we can find that the equivalent initial launch angle of the generated Airy-like beam is increased with the Airy fiber length. Thus, the first type of phased array modulation is based on changing the fiber length can be implemented to generate an Airy beam. As increasing the fiber length, the transverse shift of the output beam is enhanced during propagation, as shown in Fig. 5(f). Therefore, one can choose a suitable length of Airy fiber to generate an Airy-like beam with an expected phase distribution and an expected equivalent initial launch angle that can cause the main lobe following a certain curved trajectory. Notably, the generated Airy-like beam has almost the same acceleration ability as the ideal Airy beam. As a result, the propagation trajectory of the Airy-like beam (see the solid line marked D in Fig. 5(f)) matches pretty well with the ideal Airy beam (see the dashed line in Fig. 5(f)). From Fig. 5(f), we note that a Gaussian-like beam is generated by a Z_G_ length of the Airy fiber and there is almost no deflection during propagation during short-range propagation (see the solid line marked G in Fig. 5(f)). For the output beam from the Airy fiber with 3.68 mm (Z_F_) length in the inset of Fig. 5(f), we can clearly see that the intensity of the Airy-like beam decreases rapidly during propagation until its main lobe disappears after propagating about 0.4 mm (see the solid line marked F in Fig. 5(e)). The cause of this unexpected behavior is that the optical power of the main lobe heals the side lobes and internal lobes so that it can not recovery due to too strong transverse shift, even at the beginning, the beam has a strong main lobe (see Fig. 1(a)). Therefore, by increasing the Airy fiber length, one can obtain an Airy-like beam which has enhanced capacity for the transverse shift but weak ability to remain nondiffraction. Note that at the beginning the beams from Airy fiber do not accelerate and their deflections go to the negative value, as shown in Fig. 5(f). It is because that a part of the beam energy is used to form the internal lobes so that the total power flow is along the direction of the positive X1-axis, which is just opposite to the desired acceleration direction.

### Phased-array control effect in the bent Airy fiber

As we described in the previous section, a discrete optical system is designed by using an Airy fiber with two perpendicular arrayed cores to implement transform between Gaussian beam and Airy beam through Airy amplitude and phased array modulation. In this section, we give another type of phased array modulation based on the bending effect. Unlike the previous type of phased array modulation by changing the length of a straight Airy fiber, this type is modulated by the magnitude and the direction of fiber bending.

**Figure 6:**
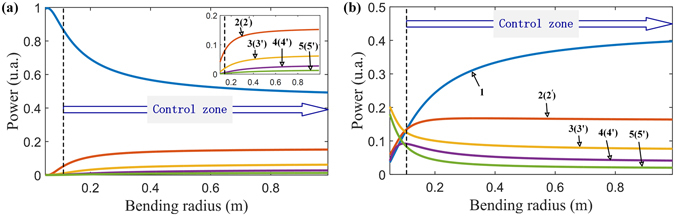
The output powers from arrayed cores of the Airy fiber as a function of bending radius. (**a**) Bending along the positive X1-aixs direction. (**b**) Bending along the negative X1-aixs direction. Note that we do not consider the bending loss for a short Airy fiber with several millimeter lengths. Inset in (**a**) shows zoomed-in partial details.

Figure 6 shows the output powers from arrayed cores of the bent Airy fiber with Z_D_ = 2.2 mm length. We can clearly see that the output powers from all arrayed cores are monotone changing with the bending radius when the bending radius is greater than 0.1 m depicted by the dashed line in Fig. 6(a) and (b). From eq. (15), we can find that the equivalent RI is inversely proportional to the bending radius. In the case of the positive bending along the X1-aixs, as the bending radius decreases the equivalent RI of side cores in two perpendicular arrayed-cores increases compared with the original without bending, resulting in weakening optical power coupling between main core and side cores. Therefore, the output powers of the main core increases but side cores’ decrease while more and more power is kept in the main core with the bending radius decreasing, as shown in Fig. 6(a). On the contrary, as the bending radius decreases more optical power from the main core is coupled into side cores. As a result, we can clearly see that such optical coupling causes the output power of the main core decreasing but side cores’ increasing, as shown in Fig. 6(b).

**Figure 7:**
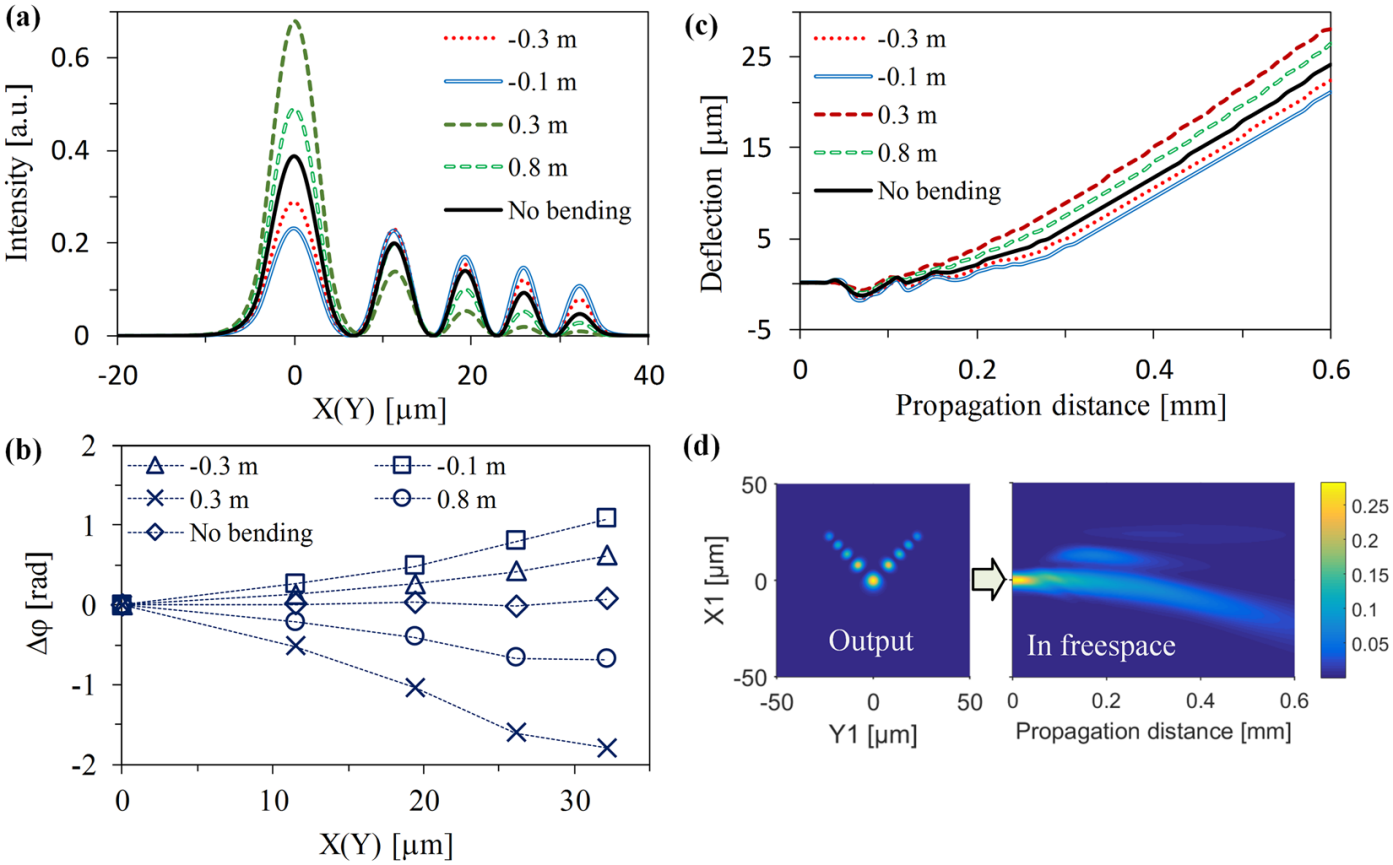
The characteristics of the output beam from the Airy fiber with different bending radius. (**a**) The intensity distributions. (**b**) The phase profiles at local intensity peaks. (**c**) Deflection of main lobes of the beams during propagation in free space. (**d**) Optical field distributions of the transverse output beam (left) and its longitudinal propagation beam (right) in free space when the bending radius of Airy fiber is –0.1 m.

**Figure 8:**
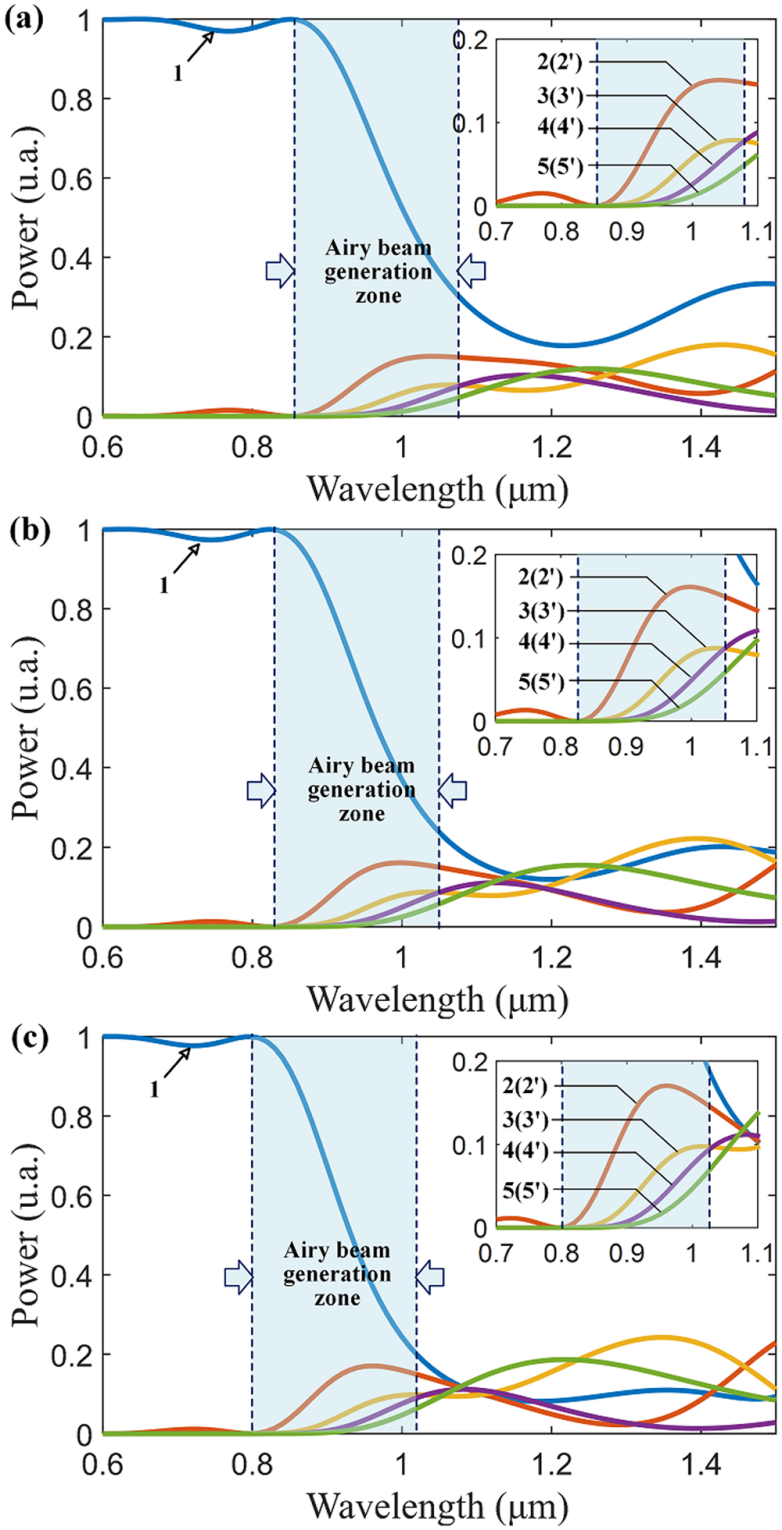
Wavelength response of Airy fiber bent along the X1-axis. (**a**) Positively bending R_b_ = 0.3 m. (**b**) No bending. (**c**) Negatively bending R_b_ = −0.3 m.

Figure 7(a) and (b) give the results of Airy amplitude and phased array modulation for a generated Airy beam from the bent Airy fiber with Z_D_ = 2.2 mm length. We can clearly see that the output beam’s intensity and phase distributions can be easily changed by bending the fiber. When the bending radius of Airy fiber is positive, there is much greater intensity for the main lobe of the output Airy beam but smaller for side lobes, as shown in Fig. 7(a). In this case, the equivalent initial launch angle (the slope of the trend line of the phase profile shown in Fig. 7(b)) is always negative so that the transverse shift is stronger than the general Airy beam from Airy fiber without bending, as shown in Fig. 7(c). On the other hand, there are much stronger side lobes but weaker main lobe when we bend the fiber along negative X1-axis. And the positive equivalent initial launch angle (see Fig. 7(b)) causes the output Airy beam has a weak transverse shift but an enhanced capacity to remain nondiffraction during propagation, as shown in Fig. 8(c) and (d). Note that there is almost propagation-invariant intensity for the main lobes of the output Airy beam and we can also clearly see that an internal lobe reborn due to self-healing, as shown in Fig. 7(d).

**Figure 9:**
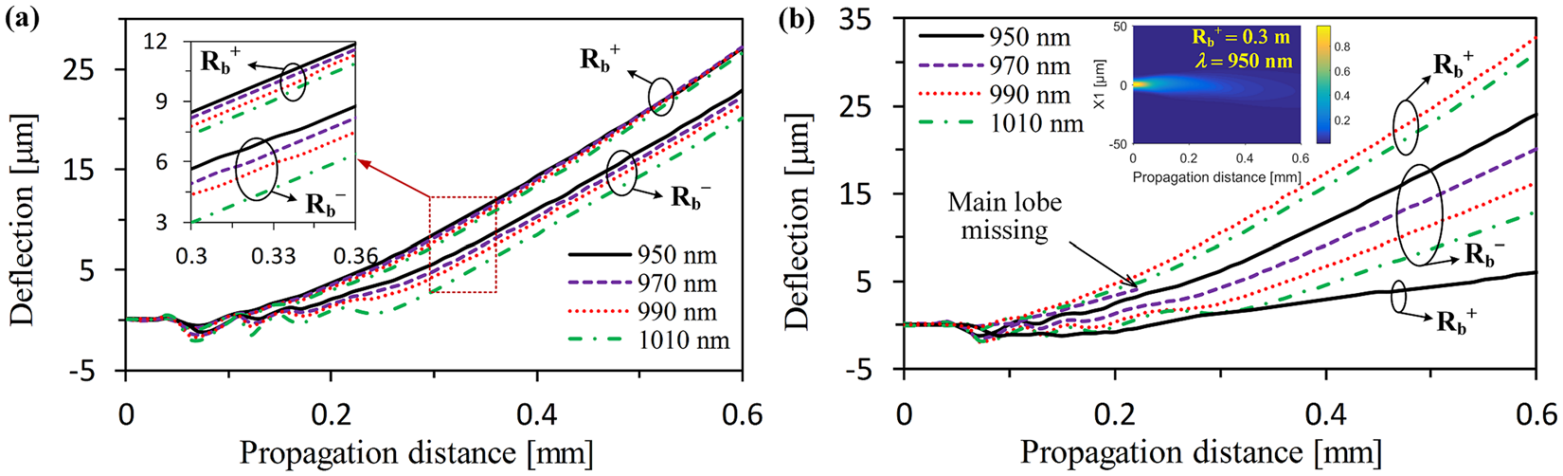
The dynamics of the generated Airy beams for different wavelength during propagation in free space. (**a**) Output beams from 2.2 mm length of the Airy fiber. The inset is corresponding to zoomed-in views. Here R_b_^+^ and R_b_^−^ are 0.3 m and −0.3 m, respectively. (**b**) Output beams from 6.6 mm length of the Airy fiber. The inset indicates the beam’s weak transverse shift due to lack of side lobes.

In order to reveal wavelength response of the Airy fiber, we calculate the output spectrum of nine cores of the 2.2 mm (Z_D_) length of Airy fiber with different bending radius, as shown in Fig. 8(a)–(c). An Airy beam generation zone is defined for evaluating the relationship between the output spectrum and the bending radius. In the Airy beam generation zone, note that the output powers of nine cores are almost monotone changing with optical wavelength. As mentioned above, from eq. (12) an Airy beam can be generated through suitable phase modulation, which can be expressed as:

1 $$\phi ={\beta }_{i}^{^{\prime} }\cdot {z}_{{\rm{0}}}=\frac{{\rm{2}}\pi }{\lambda }{n}_{i}^{^{\prime} }\cdot {z}_{{\rm{0}}},$$

where ${n}_{i}^{^{\prime} }$ is the effective RI of the supermode propagation in Airy fiber, *z*_0_ is the length of the fiber. From eq. (1), we can find that the phase modulation depends on the optical wavelength *λ*, the effective RI of the supermode ${n}_{i}^{^{\prime} }$, and the fiber length *z*_0_. For a length of the Airy fiber, the effective RI ${n}_{i}^{^{\prime} }$ is proportional to the optical wavelength *λ* for ensuring the same modulation phase *φ*. Actually, the effective RI ${n}_{i}^{^{\prime} }$ is determined by the RI distribution of fiber. Thus, we can control the effective RI ${n}_{i}^{^{\prime} }$ by changing the equivalent RI of the bent fiber in our case. And that means the equivalent RI profile changing in fiber causes the effective RI ${n}_{i}^{^{\prime} }$ increases (decreases) when we positively (negatively) curve the Airy fiber. Therefore, from Fig. 8(a)–(c), we can clearly see that the Airy beam generation zone of Airy fiber with the positive and negative bending radius is shifted to longer and shorter wavelengths, respectively. What is also quite interesting is the fact that the curved trajectory of the main lobe of the Airy-like beam from the negatively bent Airy fiber varies sharply with the wavelength, as shown in Fig. 9(a). For the negatively bent Airy fiber, however, it is not sensitive. This remarkable characteristic is named “rainbow effect” due to the transverse shift control induced by phased array modulation^23^. As the wavelength increases, the phase modulation is weakened from equation (1) so that the equivalent initial launch angle decreases, resulting in weakening the transverse shift of the beam, as shown in Fig. 9(a). And this phenomenon is more evident for the 6.6 mm (3*T*_0_/2) length of the Airy fiber, as shown in Fig. 9(b). The reason is quite obvious that the phase modulation is enhanced when the fiber length *z*_0_ increases from eq. (1). We can see that the curved trajectories of the beams from the negatively bent Airy fiber are obviously separated by wavelength manipulation in Fig. 9(b). However, the generated beams from the positively bent Airy fiber are still not sensitive. And the main lobe may vanish due to too strong transverse shift in wavelength manipulation, as shown in the dashed line of Fig. 9(b) for R_b_^+^. For a shorter wavelength, such as 950 nm, the side lobes of the generated beam could disappear and only the main lobe can be preserved. And the transverse shift of the beam is very weak (see the solid line of Fig. 9(b) for R_b_^+^). And its propagation trajectory is similar to the Gaussian beam, as shown in the inset of Fig. 9(b). In general, by changing any of the parameters, including fiber length, optical wavelength, and bending-induced refractive-index changing, one can obtain a desired Airy-like beam or Gaussian-like beam from an Airy fiber.

**Figure 10:**
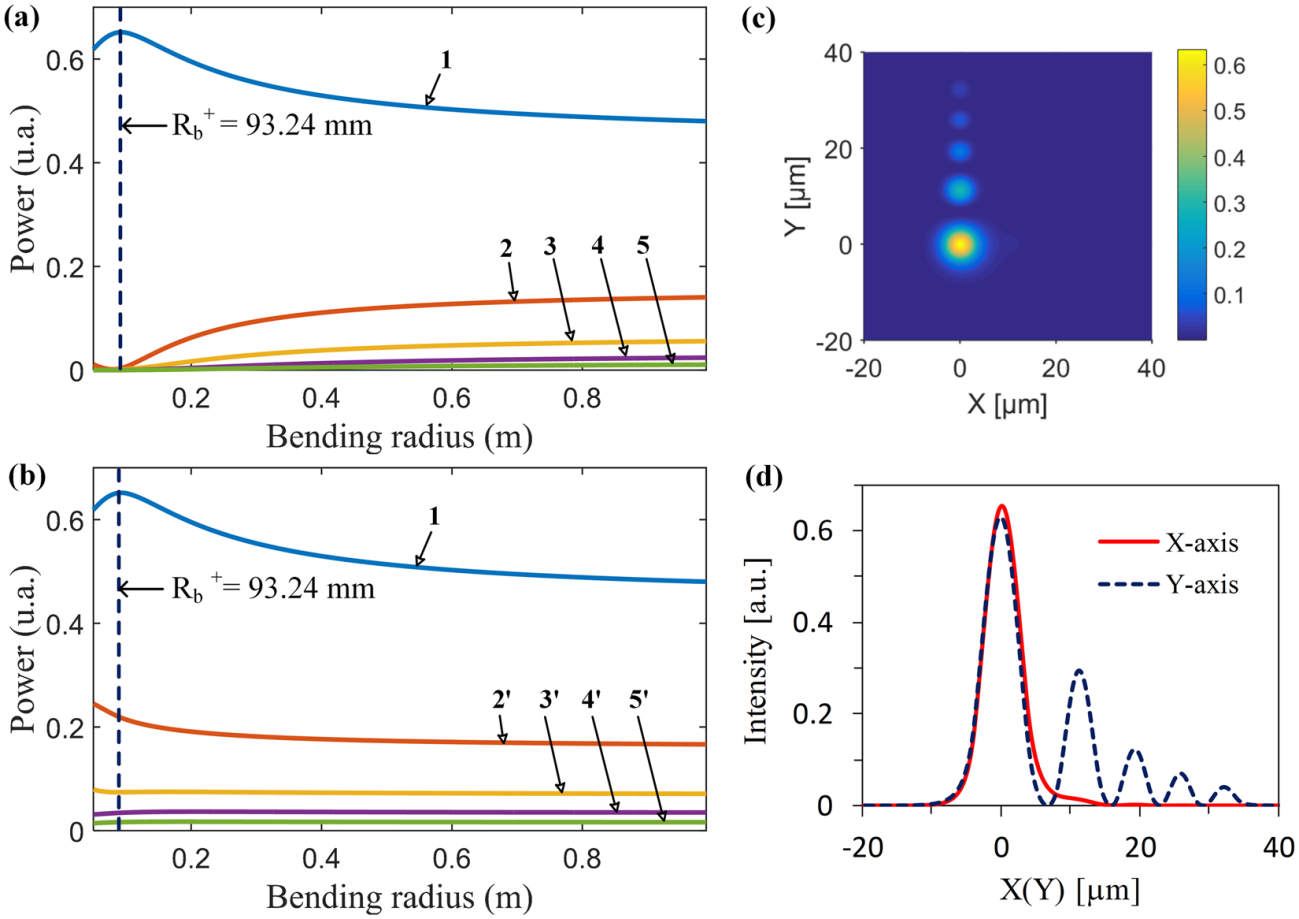
Bending an Airy fiber along the positive X-axis direction. (**a** and **b**) are the output powers of Airy fiber arrayed cores on the X- and the Y-axis as a function of bending radius, respectively. (**c** and **d**) are the transverse output field and its intensity profiles along the X- and the Y-axis, respectively, when the bending radius is 93.24 mm. Here R_b_^+^ is the bending radius along the positive X1-axis.

From eq. (15), we can find that the equivalent RI of the bent Airy fiber is directly affected by the angle ${\tilde{\theta }}_{b}$ between the arrayed-core and the bending direction. Therefore, we can obtain asymmetric RI distribution of two perpendicular arrayed cores of an Airy fiber by changing bending direction. Figure 10(a) and (b) respectively give the output power profiles of two perpendicular arrayed cores along the X- and the Y-axis when we curve the Airy fiber along the positive X-axis direction. In this case, the equivalent RI along the X-axis increases but is invariant along Y-axis. As a result, the output powers of the arrayed-core along the X- and the Y-axis are different. The former (except the output power in the main core 1, which is located in the origin) are much smaller than the latter, as shown in Fig. 10(a) and (b). Remarkably, a one-dimensional Airy beam is generated when the bending radius is 93.24 mm, as shown in Fig. 10(c). From Fig. 10(d), we can clearly see that the output beam has an Airy distribution along the Y axis but almost no energy in the side cores of the arrayed-core along the X-axis. Therefore, we can obtain a desirable 1D or 2D Airy beam by choosing a suitable bending radius of the Airy fiber along an appropriate direction.

## Discussion

We have provided several effective approaches to control the propagation dynamics of light in a discrete optical system of Airy fiber. The supermode theory and refractive-index equivalent method have been applied to analyze propagation properties in the straight and the bent Airy fibers. The calculated results showed that the amplitude and phased array modulation on a Gaussian beam are dependent on the fiber parameters, such as fiber length, refractive-index distribution, and the incident wavelength. By changing these parameters, we can perfectly control the wavefront of light propagation in the Airy fiber and obtain an output Airy beam with desirable abilities to remain quasi-nondiffraction propagating, self-healing and transverse accelerating. We also obtain the one-dimension Airy beam or the Gaussian beam in one same Airy fiber through parameter control. Therefore, we not only give a tool of a beam generator but also proposed a technology to control the propagation dynamics of light in a general discrete optical system. This technology might apply to other interesting areas, including integration optics and nonlinear optics.

## Methods

### Airy beam

Considering the input field distribution

2 $$\psi (x,y,z=0)={\prod }_{m=x,y}\mathrm{Ai}({s}_{m})\,\exp ({a}_{m}{s}_{m})\,\exp ({\rm{i}}{v}_{m}{s}_{m}),$$

the two-dimension (2D) finite energy Airy beam envelope expresses as follows^3^:

3 $$\psi (x,\,y,z)=\prod _{m=x,y}{u}_{m}({s}_{m},{\xi }_{m}),$$

where

4 $$\begin{array}{rcl}u({s}_{m},\,{\xi }_{m}) & = & {\rm{Ai}}\,[{s}_{m}-\frac{{\xi }_{m}^{2}}{4}-{v}_{m}{\xi }_{m}+i{a}_{m}{\xi }_{m}]\,\exp [\,{a}_{m}{s}_{m}-\frac{{a}_{m}{{\xi }_{m}}^{2}}{2}\,-{a}_{m}{v}_{m}{\xi }_{m}\\  &  & +{\rm{i}}(-\frac{{\xi }_{m}^{3}}{12}+({a}_{m}^{2}-{v}_{m}^{2}+{s}_{m})\frac{{\xi }_{m}}{2}+{v}_{m}{s}_{m}-\frac{{v}_{m}{\xi }_{m}^{2}}{2})],\end{array}$$

where Ai(*s*) represents the Airy function, ${\xi }_{m}=z/k{m}_{0}^{2}$ are normalized propagation distance, where $k=2\pi n/\lambda$ is the wavenumber of incident wavelength *λ* with the refractive-index *n* of the propagation medium. ${s}_{m}=-(m-{m}_{1})/{m}_{0}$ represents dimensionless transverse coordinate, with *m*_0_ being arbitrary transverse scales. *m*_1_ is the coordinate of the first maximum of the amplitude function of the ideal Airy field $|{\rm{Ai}}(m/{m}_{0})\,\exp$ $({a}_{m}m/{m}_{0})\,\exp (i{v}_{m}m/{m}_{0})|$, and *a*_*m*_ is the apodization rate. ${v}_{m}=-{\theta }_{m}k{m}_{0}$ is related to the initial launch angle *θ*_*m*_ of this beam, which is also corresponding to the *m* (*x* or *y*) component of the initial “velocity” along the direction of the beam acceleration bending^4^.

### The mode coupling calculation in straight Airy fiber

We employ coupled-mode theory (CMT) to study the propagation behavior in Airy fiber^24^. The coupling relation among nine arrayed cores of the Airy fiber should be described as following coupling equations

5 $$\frac{d{\tilde{a}}_{p}}{dz}=-j{\beta }_{p}{\tilde{a}}_{p}-j\sum _{q=1}^{9}{\kappa }_{pq}\,{\tilde{a}}_{q},$$

where ${\tilde{a}}_{p}={a}_{p}\,\exp (-i{\beta }_{p}z)$ denote the rapidly varying expansion coefficients of eigen mode (***E***_*p*_, ***H***_*p*_) in Core *p*, and *β*_*p*_ is propagation constant of the mode. *κ*_*pq*_(*q* ≠ *p*) is mode coupling coefficient between Core *p* and Core *q*. And *κ*_*pp*_(*q* = *p*) is the self-coupling coefficient of Core *p*. Here *κ*_*pq*_ and *κ*_*pp*_ can be defined as:

6 $${\kappa }_{pq}=\frac{\omega {\varepsilon }_{0}}{4\sqrt{{P}_{p}{P}_{q}}}{\int }_{S}[{n}_{p}^{2}(x,y)-{n}_{0}^{2}]{{\boldsymbol{E}}}_{p}\cdot {{\boldsymbol{E}}}_{q}^{\ast }\,dS,$$

7 $${\kappa }_{pp}=\frac{\omega {\varepsilon }_{0}}{4{P}_{p}}{\int }_{S}[{\bar{n}}^{2}(x,y)-{n}_{p}^{2}(x,y)]{{\boldsymbol{E}}}_{p}\cdot {{\boldsymbol{E}}}_{p}^{\ast }\,dS,$$

where $\bar{n}(x,y)$ is the RI distribution in the entire arrayed-core. *n*_*p*_(*x*, *y*) and *n*_0_ represent the RI distribution of Core *p* and the surrounding medium, respectively. The optical power carried by the eigen mode (***E***_*p*_, ***H***_*p*_) in Core *p* is given by

8 $${P}_{p}=\frac{1}{2}{\int }_{S}({{\boldsymbol{E}}}_{p}\times {{\boldsymbol{H}}}_{p}^{\ast })\cdot {{\boldsymbol{u}}}_{z}\,dS$$

where ***u***_*z*_ is the unit vector along the z-axis. Considering the matrix notation of eq. (5)

9 $$\frac{d}{dz}\tilde{A}(z)=-jM\tilde{A}(z)$$

where $\widetilde{A}(z)={[{\tilde{a}}_{1},{\tilde{a}}_{2},\cdots ,{\tilde{a}}_{9}]}^{T}$. One can obtain the eigenvalues ${\beta }_{i}^{^{\prime} }$ and eigenvectors ***V***_*i*_ (column vector) of the coupling matrix *M*. As a result, the transverse electric field of supermodes of arrayed-core can be expressed as

10 $${{\boldsymbol{E}}}_{i}^{^{\prime} }=[\begin{array}{cccc}\frac{{{\boldsymbol{E}}}_{1}}{\sqrt{{P}_{1}}} & \frac{{{\boldsymbol{E}}}_{2}}{\sqrt{{P}_{2}}} & \cdots  & \frac{{{\boldsymbol{E}}}_{9}}{\sqrt{{P}_{9}}}\end{array}]\cdot {{\boldsymbol{V}}}_{i}.$$

Supposing only the fundamental mode *LP*_01_ (Gaussian beam) of the central Core 1 as input (see Fig. 1(a)), each amplitude of supermodes of arrayed-core can be solved by eq. (9):

11 $${[\begin{array}{cccc}{\tilde{a}^{\prime} }_{1} & {\tilde{a}^{\prime} }_{2} & \cdots  & {\tilde{a}^{\prime} }_{9}\end{array}]}^{T}={[\begin{array}{cccc}{{\boldsymbol{V}}}_{1} & {{\boldsymbol{V}}}_{2} & \cdots  & {{\boldsymbol{V}}}_{9}\end{array}]}^{-1}\cdot {A}_{0}^{T}.$$

here ${A}_{0}=[1,0,\cdots ,0]$ is 1 × 9 initial condition matrix in our case. From eqs (10) and (11), we obtain the total electric field along arrayed-core:

12 $${{\boldsymbol{E}}}_{t}=\sum _{i=1}^{9}{\tilde{a}^{\prime} }_{i}\cdot {{\boldsymbol{E}}}_{i}^{^{\prime} }\cdot \exp (-j{\beta }_{i}^{^{\prime} }z)$$

### Mode fields calculation in the bent Airy fiber

**Figure 11:**
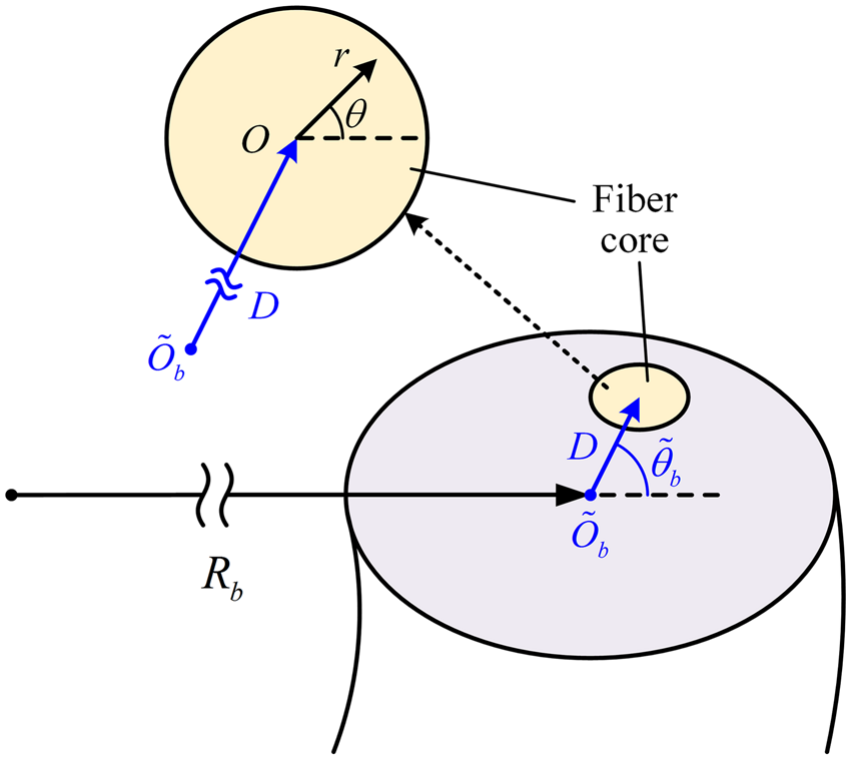
The bent fiber model. A local polar coordinate system (${\tilde{r}}_{b}$, ${\tilde{\theta }}_{b}$) for the bent fiber is introduced. And The center of the bent fiber with a bending radius *R*_*b*_ is set as the coordinate origin ${\tilde{O}}_{b}$. The radial direction is along the direction of bending. The fiber core center is located at the point (*D*, ${\tilde{\theta }}_{b}$), which is the origin *O* of another local polar coordinate system (*r*, *θ*) for fiber core.

To analysis the optical characteristics in a bent fiber, we introduce a simplified approximation model in Fig. 11. The *z*-components of the electromagnetic field (${\tilde{E}}_{{\rm{p}}}$, ${\tilde{H}}_{{\rm{p}}}$) guided in Core *p* and cladding regions of the bent fiber (see Fig. 11) can be expressed in terms of cylinder functions in the coordinate system *r*, *θ* ^25, 26^:

13 $$[\begin{array}{c}{\tilde{E}}_{p,z}\\ {\tilde{H}}_{p,z}\end{array}]=\{\begin{array}{c}\begin{array}{cc}[\begin{array}{c}{C}_{1}\\ {C}_{2}\end{array}]\,{J}_{1}({\int }_{0}^{r}\tilde{U}(r)dr)\,\exp (i\theta ) & (0\le r\le {r}_{p})\end{array}\\ \begin{array}{cc}[\begin{array}{c}{C}_{3}\\ {C}_{4}\end{array}]{K}_{1}({\int }_{0}^{r}\tilde{W}(r)dr)\,\exp (i\theta ) & (r > {r}_{p})\end{array}\end{array},$$

where *J*_1_ and *K*_1_ are the first-order Bessel and modified Hankel functions, respectively. And we define the equivalent wave numbers $\tilde{U}$ and $\tilde{W}$ in Core *p* and its surrounding cladding of the bent fiber along the transverse direction as

14 $$\{\begin{array}{c}\tilde{U}=\sqrt{{k}_{0}^{2}\cdot {\tilde{n}}_{p}^{2}(r,\theta )-{\tilde{\beta }}_{p}^{2}}\\ \tilde{W}=\sqrt{{\tilde{\beta }}_{p}^{2}-{k}_{0}^{2}\cdot {\tilde{n}}_{0}^{2}(r,\theta )}\end{array}.$$

Here, the equivalent RI of Core *p* and cladding ${\tilde{n}}_{p}(r,\theta )$ and ${\tilde{n}}_{0}(r,\theta )$ can be described as a corresponding straight fiber with an effective RI distribution after conformal mapping^27^:

15 $$\{\begin{array}{c}{\tilde{n}}_{p}(r,\theta )={\tilde{n}}_{p}(0,0)\cdot \exp (\frac{x}{{R}_{b}})\approx {\tilde{n}}_{p}(0,0)\cdot (1+\frac{r}{{R}_{b}}\,\cos \,\theta )\\ {\tilde{n}}_{0}(r,\theta )={\tilde{n}}_{0}(0,0)\cdot \exp (\frac{x}{{R}_{b}})\approx {\tilde{n}}_{0}(0,0)\cdot (1+\frac{r}{{R}_{b}}\,\cos \,\theta ).\end{array}$$

In eq. (15), the first-order approximation is used for considering a micro bend (*x* ≪ *R*) in our case. And in the polar coordinate system $({\tilde{r}}_{b},\,\,\,{\tilde{\theta }}_{b})$, the equivalent RI of Core *p* and cladding ${\tilde{n}}_{p}(0,0)$ and ${\tilde{n}}_{0}(0,0)$ at the core center $(D,\,\,\,{\tilde{\theta }}_{b})$, and the equivalent propagation constant ${\tilde{\beta }}_{p}$ are respectively given by

16 $$\{\begin{array}{c}{\tilde{n}}_{p}(0,0)\approx {n}_{p}\cdot (1+\frac{D}{{R}_{b}}\,\cos \,{\tilde{\theta }}_{b})\\ {\tilde{n}}_{0}(0,0)\approx {n}_{0}\cdot (1+\frac{D}{{R}_{b}}\,\cos \,{\tilde{\theta }}_{b})\\ {\tilde{\beta }}_{p}\approx {\beta }_{p}\cdot (1+\frac{D}{{R}_{b}}\,\cos \,{\tilde{\theta }}_{b})\end{array}.$$

Based on the relationship between transverse and longitudinal fields in axially symmetric optical fibers^28^, the transverse components (${\tilde{E}}_{p,x}$, ${\tilde{E}}_{p,y}$, ${\tilde{H}}_{p,x}$, ${\tilde{H}}_{p,y}$) of electromagnetic fields guided in bent fiber also can be obtained by substituting the primary wave numbers *U* and *W* in straight fiber with their equivalent values $\tilde{U}$ and $\tilde{W}$ in bent fiber. Further, we similarly use the equivalent factors of the bent fiber to instead of the original factors of the straight fiber in eqs (5–7) for solving the mode coupling in the bending Airy fiber.
